# Effectiveness of manual therapy compared to usual care by the general practitioner for chronic tension-type headache: design of a randomised clinical trial

**DOI:** 10.1186/1471-2474-10-21

**Published:** 2009-02-12

**Authors:** René F Castien, Daniëlle AWM van der Windt, Joost Dekker, Bert Mutsaers, Anneke Grooten

**Affiliations:** 1 Healthcare Center Haarlemmermeer, Hoofddorp, the Netherlands; 2 EMGO Institute, VU-University Medical Center, Amsterdam, the Netherlands; 3 Primary Care Musculoskeletal Research Centre, Keele University, Keele, Newcastle-under-Lyme, Staffordshire, UK; 4 Avans Hogeschool, Breda, the Netherlands; 5 Department of Rehabilitation Medicine, VU-University Medical Center, Amsterdam, the Netherlands

## Abstract

**Background:**

Patients with Chronic Tension Type Headache (CTTH) report functional and emotional impairments (loss of workdays, sleep disturbances, emotional well-being) and are at risk for overuse of medication. Manual therapy may improve symptoms through mobilisation of the spine, correction of posture, and training of cervical muscles.

We present the design of a randomised clinical trial (RCT) evaluating the effectiveness of manual therapy (MT) compared to usual care by the general practitioner (GP) in patients with CTTH.

**Methods and design:**

Patients are eligible for participation if they present in general practice with CTTH according to the classification of the International Headache Society (IHS).

Participants are randomised to either usual GP care according to the national Dutch general practice guidelines for headache, or manual therapy, consisting of mobilisations (high- and low velocity techniques), exercise therapy for the cervical and thoracic spine and postural correction. The primary outcome measures are the number of headache days and use of medication. Secondary outcome measures are severity of headache, functional status, sickness absence, use of other healthcare resources, active cervical range of motion, algometry, endurance of the neckflexor muscles and head posture. Follow-up assessments are conducted after 8 and 26 weeks.

**Discussion:**

This is a pragmatic trial in which interventions are offered as they are carried out in everyday practice. This increases generalisability of results, but blinding of patients, GPs and therapists is not possible.

The results of this trial will contribute to clinical decision making of the GP regarding referral to manual therapy in patients with chronic tension headache.

## Background

The 1-year prevalence of Chronic Tension-Type Headache (CTTH) is about 2–5% in the general population. In half of the CTTH cases, headache-related impairment in work performance is reported. [1,2] In addition to considerable impact on daily functioning and work participation, CTTH is a risk factor for overuse of analgesic medication [3]. Only about 20% of the CTTH patients seek medical care for their headache. This low consultation rate may be explained by insufficient information on the effectiveness of treatments or by previous negative health care experiences.[1,4]

In primary care treatment for patients with CTTH is often provided by the general practitioner (GP). The Dutch national general practice guideline for the management of headache describes diagnostic and therapeutic algorithms, consisting mainly of reassurance, lifestyle advice and medication.[5] The effectiveness of this guideline for patients with CTTH has not been investigated.

The pathogenesis of CTTH remains unclear. Pathophysiological theories considering central and peripheral pain mechanisms are described and have been discussed in the literature. [6] In recent research a correlation between CTTH and impairment of the cranio-cervical musculoskeletal function (forward head position, trigger points trapezius muscle, neck mobility) has been demonstrated [7-10] In combination with results obtained in previous studies the present data support the hypothesis that improvement of the cranio-cervical musculoskeletal function by a manual therapy intervention (postural correction, mobilisation cervical spine, and training of cervical muscles) may be an important factor to modify central or peripheral pain mechanism in CTTH. [11-15]

Three randomized clinical trials have investigated the effectiveness of manual therapy in patients with CTTH and reported benificial effects.[16-18]. However, because of variation in inclusion criteria, treatment techniques (high-, low velocity mobilization, exercises, traction), and small sample sizes there is insufficient evidence to support the use of manual therapy in the treatment of CTTH. Well-designed clinical trials are recommended to provide more substantial evidence for the effectiveness of manual therapy. [19,20]

## Design

We aim to conduct a pragmatic, multicentre, randomised clinical trial, assessing the effectiveness of manual therapy (MT) compared to usual GP care in patients with CTTH. We have used the guidelines of the International Headache Society (IHS) for the design of randomised clinical trials for headache to develop the randomisation procedure, outcome measurements and statistical analysis.[21] The procedures and design of this study are approved by the Medical Ethics Committee of the VU University Medical Center in Amsterdam, The Netherlands. (Trial registration number TR 1074)

### Study population

Participating primary healthcare centers and GPs in an urban area in the Netherlands, will invite patients with headache to participate in the trial.

Patients between 18 and 65 years of age are invited if they have CTTH according to the classification of headaches of the IHS [22]: headache occuring on at least 15 days on average per month for a period of more than 3 months (≥ 180 days a year) and lasts for hours or may be continuous. The headache has at least one of the following characteristics: 1. bilateral location, 2. pressing/tightening (non pulsating) quality, 3. mild or moderate intensity, not aggravated by normal physical activities such as walking or climbing stairs; and both of the following: 1. no more than one of photofobia, phonophobia or mild nausea, and 2. neither moderate or severe nausea nor vomiting. Participants should be able to read and write Dutch.

Exclusion criteria include reumatoid arthritis, suspected malignancy, pregnancy, intake of either triptans, ergotamines or opioids on ≥ 10 days/month or simple analgesics on ≥ 15 days/month on a regular basis for ≥ 3 months, and having received manual therapy treatment in the 2 months before enrolment into the study.

**Figure 1:**
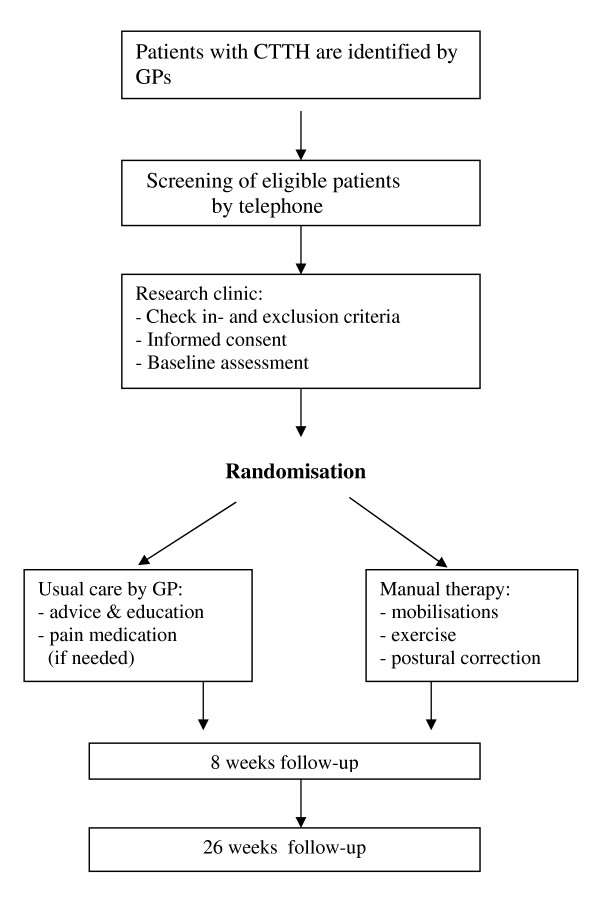
**Flow chart, representing the design of the trial on Chronic Tension Type Headache (CTTH)**.

After the GP has seen a patient with CTTH the patient receives an information letter about the trial. If the patient is willing to participate after reading the information he or she can contact the research centre. A researcher will screen interested patients by telephone and make an appointment to check inclusion and exclusion criteria, and complete the informed consent procedure. After written informed consent has been obtained, the baseline measurement is carried out. The design of the trial is explained in Figure 1.

### Baseline assessment

Table 1 shows the outcome measures and the time points at which they are assessed. At baseline we will collect additional information on demographic variables including age, gender, date of birth, education and occupation. The patient will also be asked to score expectations regarding the effectiveness of treatment on a 7-point rating scale (no result at all to very good result). In a standardised history taking procedure including the two-week headache-diary, the diagnosis of CTTH according to the diagnostic criteria of the IHS guideline will be confirmed.[22]

**Table 1:** Summary of data collection.

	**Baseline**	**After 8 weeks**	**After 26 weeks**	**Measurement**
Sociodemographics	X			Single items

Treatment expectations	X			7-point rating scale

**Primary outcome measures**				

Headache frequency	X	X	X	Headache diary

Medication use	X	X	X	Headache diary

**Secondary outcome measures**				

Headache pain intensity	X	X	X	10 points scale

Headache Impact on daily life	X	X	X	HIT-6 HDI

Active range of movement Cervical spine	X	X	X	CROM

Algometry M. Trapezius and suboccipital muscles	X	X	X	Algometer

Craniocervical angle	X	X	X	Digital photo

Endurance neck flexor muscles	X	X	X	Neck flexor endurance test

Use of health care resources			X	Checklist

### Randomisation

Randomisation will take place after baseline measurement by the research assistant. Before the start of the trial a random sequence has been composed using computer-generated random numbers. Allocation is carried out by the research assistant who has not been informed about the random sequence, by giving the patient a numbered and sealed envelope.

The patient will open the envelope in the presence of another independent administrative assistant, who will subsequently make an appointment for the first treatment session either by the patient's own GP, or by one of the participating manual therapists.

### Blinding

GPs and manual therapists cannot be blinded for treatment allocation, but will not be informed about the results of outcome measurements. The research assistant is kept blind for the patient's treatment allocation.

Data collection and administration will by carried out by an independent data assistent. The researcher is involved in the statistical analysis, but the analysis and interpretation of the findings will be audited and verified by an independent statistician.

### GP intervention

Patients will be treated by the GP according to the national clinical guideline for the management of headache [5]. According to this guideline the GP will provide information, reassurance and advice and will discuss the benefits of lifestyle changes. If necessary, GPs may prescribe analgesics or non- steroid anti-inflammatory drugs (NSAID) or change current pain medication.

### Manual Therapy intervention

MT treatment will include a combination of mobilisation of the cervical and thoracic spine, exercises and postural correction based on the management of cervicogenic headache. [23] Spinal mobilisations will consist of low and/or high-velocity cervical and thoracic joint mobilization and manipulation techniques. Therapeutic exercises consist of low-load craniocervical muscle endurance exercises and correction of sitting and standing posture. The participating MT's are registrated MT's and member of the national association of manual therapists. They have an average experience of 10 years as manual therapist and have completed the McKenzie B-course on the cervical spine. In two meetings the MTs have been trained in the treatment protocol, they have received a manual and patient-booklets with home exercises.

Depending on the patient's condition the MT can decide what type of techniques and exercises will be selected from the protocol. The MT will make a report of the treatment modalities used in each session.

The MT intervention is restricted to a maximum of 9 sessions (each 30 minutes) in 8 weeks after randomisation.

### Primary outcome measures

The follow-up measurements will take place by a blinded research assistant immediately after the 8 weeks treatment period and after 26 weeks (long term follow-up). Two weeks before each measurement the patients receive and complete a two-week headache diary. The primary outcome measures are 1) the frequency of days with headache, and 2) use of pain medication (no. of doses NSAIDs or simple analgesics). Registration over a two week period is considered to be sufficient.[24]

### Secondary outcome measures

The secondary outcome measures include:

* Headache pain intensity measured on a 10 point numerical rating scale (0 = no pain, 10 = most severe pain).

* The impact of headache on daily life will be scored by the patient using the Headache Disability Inventory (HDI) and the Headache Impact Test-6 (Hit-6). The HDI includes 25 questions on physical and emotional functioning with three possible response options: no = 0 points, sometimes = 2 points, yes = 4 points. A total score is computed by summating all scores, resulting in an individual HDI score ranging from 0 (no disability) to 100 (severe disability). A decrease in the total HDI of ≥ 16 points is considered to be a significant improvement. The test-retest reliability of the total score has been shown to be adequate (Pearson *r *0.76 for 1 week; *r *= 0.83 for 6 weeks) [25]

* The Headache Impact Test (HIT-6) consists of 6 items (pain intensity, social functioning, role functioning, vitality, cognitive functioning and psychological distress) each with 5 response options; never: 6 points, rarely: 8 points, sometimes: 10 points, very often: 11 points, always: 13 points, with a total score ranging from 36 to 78 points. Internal consistency (Cronbach alpha: 0.89) and test-retest reliability (ICC ranging from 0.78 to 0.90) have been demonstrated to be good.[26] The HIT-6 is able to differentiate between mild, moderate and severe headache. A between-group difference in HIT 6 change score of 2.3 points over time among patients with chronic daily headache reflects improvement in headache that may be considered to be clinically significant. [27]

* The active range of movement in flexion, extension, right and left rotation and right and left lateroflexion of the cervical spine with the patient in a seated position will be measured by the research assistant with the CROM-device. The intra- and intertester reliability have been shown to be good (ICC. > 0.80). [28]

* Algometry on the trapezius descendens and the suboccipital muscle will be performed with a Wagner FDK algometer with a 3.0 kg/cm pressure at four points at the left and right side: two points on the upper trapezius muscle and two points on the suboccipital muscle. Patients will rate the severity of pain on a 0–10 point NRS scale (0 = no pain, 10 most severe pain). Scores for each pressure point will be summated into a total score ranging between 0 and 80 points. Mechanical pressure algometry has been described by several authors as a valid measurement for pain pressure treshold for the trapezius muscle and has a good to excellent intertester- (ICC 0.70–0.91), intratester reliability (ICC 0.84–0.88) and a intra-individual coefficient of variation of 18.5% at 1 week test-retest. [29-31]

* Endurance of the neck flexor muscles will be scored as the number of seconds the patient can raise his head from the table when lying on his/her back. In a study of the neckflexor endurance test among subjects without neck pain Harris et al. reported good to excellent intratester reliability (ICC 0.82–0.91) and moderate intertester reliability (ICC 0.67–0.78).[32]

* A lateral digital picture with a digital HP R707.5 camera will be taken in a seated and standing position to measure the craniocervical angle. Recently van Niekerk et al. evaluated the criterion validity of photographic measurement compared with a digital radiographic device (LODOX) for assessing the craniocervical angle in sitting position among high school students (Pearson *r *0.89).[33] The reliability of photographic measurement of the craniocervical angle has been reported to be good in two studies(ICC >0.86). [33,34]

* Additional use of health care resources (including GP, psychologist, physiotherapist, acupuncture) will be reported by the patient at 26 week follow-up by completing a checklist. The patient will also be asked to report perceived improvement following treatment on a 7 point scale. (0 = much worse to 6 = much better).

### Sample size

In a pilot study the 2 weeks headache diary showed an average of 11 days with headache in both treatment groups at baseline. After 8 weeks the frequency of days with headache in the GP group was reduced to 7 days, in the MT group to 3 days. In the full trial we aim to detect a difference in reduction of at least 3 days (SD 5) between both groups. To detect this difference with a one-sided significance level of 0.05, and power of 0.80 we have to include at least 35 patients in each treatment group. The participants in the pilot study reported taking on average 2 doses of NSAID or analgesics per 2 weeks. With a sample size of 35 patients per group we can detect a difference of at least 0.5 (SD 0.8) doses per 2 weeks between the groups. With a calculated loss of participants in the full trial of 15%, this trial attempts to enroll forty-two patients with CTTH in each treatment groups (GP, MT).

### Statistical analysis

Baseline comparability will be investigated by descriptive statistics to examine whether randomisation was successful. For each patient, the change between baseline and follow-up will be calculated for all primary and secondary outcome measures. The statistical analysis will be performed according to the intention-to-treat principle.

Between group differences and 95% confidence intervals will be calculated, and tested using the Student t-test in case of normal distributions. Non paramaratric testing will be used for non-normal distributions. In addition, a per-protocol analysis will be performed, analysing only those patients with no serious protocol deviations. Comparing the results of the intention-to-treat and the per-protocol analysis will indicate if and to what extent protocol deviations might have influenced the results. Multivariate regression analysis will be conducted to examine the potential influence of differences in baseline characteristics on outcome.

If results on primary outcomes show normal distributions we will compute effect sizes (standardised mean differences) as the mean difference between groups over the pooled standard deviation. Effect sizes will be rates as follows: small (0.2–0.5), medium (0.5–0.8) or large (>0.8).

### Feasibility of the study design

A pilot study was conduced between June 2006 and December 2006 to evaluate the feasibility of the measurements, randomisation-procedure and treatment protocols (33). The recruitment of participants for this pilot-study took place in two primary health-care centers in The Netherlands. A total of 20 patients were randomised to either the GP or the MT intervention group. Thirty-one patients who had a strong preference for the manual therapy intervention and could not be randomised were asked to participate in a parallel cohort-study. In this study similar baseline and follow up measurements were conducted.

The results of the pilot study showed that the procedures were feasible. The research-assistants, general practitioners and manual therapists reported having no problems to adhere to the guidelines and protocols for measurements and treatment. In order to include a total of 80 patients over a period of one year, 32 GPs and 4 MTs have been recruited to participate in the full trial.

## Discussion

We have described the design of a RCT to evaluate the effectiveness manual therapy compared to GP usual care for patients with CTTH. Both approaches are commonly used: most GPs will consult recommendations by the national clinical guideline when managing patients with headache. The manual therapy intervention is based on a treatment protocol developed in consensus with the participating MTs, and consists of commonly used mobilisation techniques and exercises. This pragmatic design will increase the external validity of the results of this trial. The MT treatment is assumed to improve cervical and thoracic spine movement and function, leading to a decrease in the frequency of headache-days. The education and exercise-training in this program is focused on self-mangement and postural correction, and aims for a sustained long-term effect. Although van Ettekoven et al [34] reported beneficial effects of a craniocervical training program for patients with CTTH, it still remains unclear what mechanisms may explain these effects. Measurement of the neckflexor muscle endurance, active range of motion of the cervical spine and the craniocervical angle in our study will provide more information on assumed processes during treatment.

This trial has a few limitations. The first limitation is the limited possibilities for blinding. Double blinding procedures in a pragmatic study cannot be obtained, and it is not possible to blind the participating GPs, MTs and patients for intervention. We do hope to reduce the risk of information bias by using standardised procedures and assessment by a blinded research assistant. The second limitation of this pragmatic trial concerns the difference in time spent on the patients' treatment by the GP and MT. It is unclear to what extent this time-factor will attribute to the overall effect of manual therapy.

Inclusion of a sufficient number of eligible patients for the RCT will be the most difficult element of this study. Rasmussen et al described a low consultation rate in patients with CTTH: only twenty percent will consult their GP [4]. The GPs will have to identify these patients during office hours and inform them about the trial. This method of recruitment has been reported to be associated with low recruitment rates.[35] In order to optimize the inclusion of patients the GPs and healthcare centers receive 'newsletters' and visits from the researcher on a regular basis to obtain the full participation in the trial.

The pilot study demonstrated a preference for manual therapy in the majority of patients. For patients who do not consent to randomisation we will conduct a parellel cohort-study alongside the trial to monitor outcome this group of patients. Expectations regarding the result of treatment will be asked for all participants in both trial and cohort, in order to estimate the potential influence of these expectations on outcome.

To publish an article of a study design has some advantages. Publication bias can be prevented whereby only studies producing positive results are more likely to be published.[36] It also offers an opportunity to reflect critically on the study design, independently of the results.

In this trial we will evaluate and compare two treatment protocols (GP, MT) that reflect 'usual care' for patients with CTTH. Therefore, the results of this pragmatic trial will contribute to clinical decision making by the GP in patients with CTTH, providing information on the potential benefits of a referral for manual therapy in primary care in the Netherlands.

## Competing interests

The authors declare that they have no competing interests.

## Authors' contributions

RC wrote the manuscript. Critical revision of the manuscript and contribution to the study design and statistical analyses by DvdW, JD and BM. RC, DvdW, JD and AG participate in the coordination of the study. All authors read and approved the final manuscript.

## Pre-publication history

The pre-publication history for this paper can be accessed here:


